# Testing an educational intervention to enhance resilience and self-efficacy among schoolteachers in Karachi Pakistan

**DOI:** 10.1186/s13104-024-06683-1

**Published:** 2024-01-15

**Authors:** Tania Nadeem, Sahar Hamid, Shireen Shehzad Bhamani, Shahina Pirani, Farida Bibi Mughal, Asheena Shahid, Nargis Asad

**Affiliations:** 1 Department of Psychiatry, Aga Khan University, Stadium road, 74800 Karachi, PO Box 3500, Pakistan; 2 Social Sciences and Liberal Arts Institute of Business Administration, Karachi, Pakistan; 3 School of Nursing and Midwifery, Aga Khan University, Karachi, Pakistan; 4 Institute of Business Administration, Karachi, Pakistan

**Keywords:** Resilience, General self-efficacy, Schoolteachers, Intervention, Pakistan

## Abstract

**Objective:**

A pilot study using a pre-post interventional design, was carried out to evaluate the effectiveness of a resilience-based training workshop on the self-efficacy and resilience of schoolteachers in a peri-urban area of Karachi, Pakistan. Four in person workshops were held at the school’s location during January to June 2022.

**Results:**

A total of 14 teachers participated in the workshop. The effectiveness of interventions was evaluated by assessing self-efficacy and resilience in pre- and post-teaching modules. The Wilcoxon Signed Rank Test determined a significant difference between the pre- to post-module scores of Self Efficacy (*p* = 0.016) and resilience (*p* = 0.006). The pre-median scores with IQR of Self-Efficacy were 28 (10), and Resilience scores 76 (12); and post-scores for Self-Efficacy and Resilience scores increased to 35 (5.5) and 88 (14) respectively. This indicated significant improvement in general self-efficacy and resilience skills after four weeks of training. This pilot study showed that building knowledge regarding mental health struggles in students and oneself, learning ways to cope with stress and manage student behavior, and forming a peer support system are crucial in building self-efficacy and resilience in teachers.

## Introduction

Teachers can improve resilience in their students, which can ultimately improve social, academic, and emotional outcomes of these young individuals. However, to achieve this goal, the teachers themselves need help in building their capacity to thrive in difficult conditions.

### Resilient teachers

Resilience is defined as bouncing back from a difficult situation. Responding in a positive manner to problematic circumstances can show resilient behavior [1]. Resilience is not innate or fixed, rather it can be acquired or learned. It protects individuals from negative mental health issues such as depression, anxiety, helplessness, hopelessness, and fear [2]. The foundation of resilience is to promote positive psychological well-being, minimize the impact of risk, and enhance the protective factors that increase a person’s ability to deal with the challenges of life [2]. Resilient teachers can recognize and manage their own stressors, communicate effectively, and tackle different challenges in their life and the education sector [3]. They can communicate empathically to understand problems, find solutions, and provide a safe environment for students to flourish. In addition, resilience helps provide more job satisfaction, reduce burn out or teacher attrition and promotes motivation in teachers too [4]. Ultimately, a resilient teacher can help improve a student’s emotional well-being alongside their learning in the classroom [5]. A resilient teacher is seen to be more likely to have a strong allegiance to their community and better able to cope with uncertainty. Resilience can produce a stronger sense of purpose which can help a person cope with negative challenges and allow these experiences to help one grow as an educator. Therefore, it can help the teachers in keeping a growth mindset where they can look at negative experiences as important moments of learning and improvement [6].

Teacher resilience does not appear to be an intrinsic or stable skill, it is a skill that can be learned and acquired over time and can fluctuate depending on the situation [7, 8]. Unfortunately, teachers are often not trained to face the challenges of their jobs [9]. They must manage their school responsibilities, student’s education, and welfare, while taking care of their own family, finances, and mental health. Additionally, they must be aware of common psychiatric disorders in children that can affect academic performance, and the impact of environmental stressors on a students’ behavior and studying capacity. Consequently, focusing on teacher training to build resilience, promote peer camaraderie and improve their skill as an academic, is essential [10]. The concept is that if a teacher is resilient, that will come through in their classroom, possibly helping in inculcating more resilience in students as well.

### Self-efficacy in teachers

Self-efficacy, which shows belief in one’s skills to achieve goals, is another trait that can help build resilience in teachers. Self-efficacy helps a teacher feel more confident about one’s skill and encourages them to leave their comfort zone and do more [11]. It helps teachers believe in their potential to perform, come up with creative solutions to problems and develop higher levels of commitment. Building knowledge and providing a supportive community are both factors that build self-efficacy. Therefore, resilience and self-efficacy are two positive traits that encourage each other.

### How do resilient and self-efficacious teachers impact students?

Teachers can be key allies in helping students develop. A significant portion of time is spent by teachers and children in schools, approximately 120 h a month. Teachers play a massive role in not only the academic growth of children but also in their socio-emotional development. They help children in building self-esteem, communication skills, and coping mechanisms. They can be an important member of a child’s support circle. The National Child Traumatic Stress Network (NCTSN) recognizes that teachers can aid children struggling with emotional, behavioral, and academic difficulties because of Adverse Childhood Experiences (ACEs) [12].

## Objectives

The objective of the study was to evaluate the effectiveness of a resilience based training workshop on the self-efficacy and resilience of schoolteachers in a peri-urban setting in Karachi, Pakistan. This was a pilot study conducted during January to June 2022. Four in person workshops were held at the school’s location. These workshops focused on helping teachers recognize their own struggles and deal with their stress effectively. It aimed to help improve reflection skills so teachers can be aware of their own strengths and weaknesses, allowing them to improve and impart education in a holistic fashion. Additionally, the workshops tried to aid a teacher’s understanding of a student’s state of mind enabling them to recognize a student’s distress and respond to it in an empathic manner. Subsequently, the aim was to improve a teacher- student bond, build trust, allow teachers to focus on student’s strengths and be more attuned to their struggles.

This pilot study can help provide a cost effective intervention targeting teachers to enable them to help children. In developing nations with limited resources, teachers can be a useful ally to promote wellness measures for children.

### Environmental background of the study setting

Pakistan is a South Asian developing nation and has a population of 220 million [13]. Islam is the major religion practiced. Around 63% of the total population resides in rural areas while 37% in urban areas. Karachi (where this study was done) is Pakistan’s largest city, having an official population of 20.3 million [13]. The city is populated with people of diverse cultural, religious, ethnic, and socio-economic backgrounds. Violence, political conflicts, and terrorism have deeply affected this city. Poor infrastructure, lack of opportunity, lack of health care and functioning schools are further dilemmas. These challenges cause unemployment, poverty, and economic disparities.

Government run school systems are riddled with corruption, mostly present only on paper. Absence of teachers who continue to get paid, political hirings where people are not trained but given a position is common. School buildings can be in a dilapidated condition, with no furniture, often used by the principal for their own purposes. Corporal punishment is common.

This study was conducted in a government school, on the outskirts of Karachi. The population is primarily of Baloch ethnicity (immigrants for financial reasons, from a neighboring province). This school had been helped by a non-governmental organization (NGO) in collaboration with the Aga Khan Development Network (AKDN), which had improved its building structure, provided furniture and worked with teachers to encourage them to be present in school and fulfil their duties. A representative of the NGO continued to work closely with the school. This school began operating in April 2015, employing 14 female teachers and 2 male teachers. These teachers were from the same ethnic background as the students. This school was currently till grade 9. It had an equal population of girls and boys. The student population came from an extremely poverty stricken background, with parental struggles around drug use, violence and incarcerations.

This location was chosen to pilot this intervention, because it represented most of the common issues faced by government schools in Pakistan and still provided a platform, where due to NGO involvement, teachers showed an interest in improving their skills. This was a typical low-income residential area of Pakistan with poor social determinants of mental health. It provided an environment which can be reflective of other areas of Pakistan or other developing nations. Another rationale for selecting this particular school was easy accessibility as it was part of the AKDN system, a partner institute to our hospital.

This paper will discuss the methodology, describe the objectives of each workshop, highlight significant results and share feedback received.

## Methods

This study employed quasi-experimental design (pre-post intervention). Quasi-experiments aim to demonstrate causality between an intervention and an outcome at the defined interval [14]. The study population included all teachers who were currently employed at this school. The inclusion criteria of study participants included all male or female teacher, currently employed at the study setting, available during the study period, and who provided written informed consent. Participants not meeting the listed criteria were excluded from the study. The study was approved by the Ethics Review Committee of Aga Khan University (Reference number 2022-6920-20362) and permission to conduct the study at school study was obtained from the school Principal. A written informed consent was also taken from all the attendees. All possible measures were taken to address anonymity, confidentiality, and respect of the participants. All participants were given identification numbers which were used throughout, and their names were not used anywhere.

### Study measures

A demographic form was used to elicit participants’ basic information including age, gender, marital status, language, education level, and years of experience.

Two outcomes were measured: resilience and self-efficacy. Wagnild & Young’s short version of Resilience scale-14 (RS-14) was used to measure the level of resilience among teachers. It consists of 14 items measured on a 7-point rating scale. The overall score on the scale was determined by summing all the items and scores ranged from 14 to 98; higher scores are indicative of a high resilience level [15]. The RS-14 is also available in Urdu language and has been used in Pakistani context [16].

Teachers’ Self-efficacy was assessed by using General Self Efficacy scale. This scale has ten-items with the response options of a 4-point Likert scale. The overall score on the scale was determined by summing all the items and scores ranged from 10 to 40; higher scores signify higher levels of self-efficacy [17]. GSE has been used in Pakistani context and it showed high internal consistency of 0.88 [18].

### Study operations

The study was divided into three phases: pre-intervention, intervention, and post-intervention.

In the pre-intervention phase, an introductory meeting was conducted with stakeholders including teachers, school management and investigators of the study. This meeting was conducted in a study setting and the information pertaining to the overall scope and objectives of the study was shared. This meeting was important to seek stakeholders’ cooperation throughout the study. Using convenient sampling, participants who were available and willing to participate in a study provided written informed consent. Self-administered questionnaire including basic demographic form, resilience and self-efficacy tools were completed by each participant as pre-test. During this phase, training modules were developed by reviewing international literature. Four modules were formulated that included: (1) Building teacher’s emotional self-awareness, (2) Understanding a child’s struggles and state of mind, (3) Building empathic communication skills, and (4) Providing psychological first aid to students.

In the intervention phase, four sessions of 1.5 h each per week were held, in person at a school (study setting). Team members included mental health professionals, academicians, and researchers. Role modeling, case discussions, reflection and peer feedback were used as teaching strategies. Handouts/post reading materials were also provided to the participants. Assessment of the knowledge was done through pre and post-test for each module that consisted of 5 multiple-choice questions. These questionnaires were developed by researchers, based on the specific training modules covered during that week.

In the post intervention phase, participants were asked to complete RS-14 and GSE tools again, as post-test. Qualitative feedback was taken on strengths and limitations of each session and recommendations for improvement were noted. Certificate of participation was awarded to participants.

### Data analysis

Analysis was performed using the IBM SPSS Statistics version 28. Descriptive statistics were computed for categorical variables by computing their frequencies and percentages. Quantitative variables were computed as median with interquartile range (IQR). Considering non normal data, Wilcoxon sign test was conducted to compare the Knowledge score before and after each module training. Moreover, pre intervention scores of our main outcomes (resilience and general self-efficacy (GSE) scores) were also compared from post intervention scores (after 4 weeks). A p-value of < 0.05 was considered significant throughout the study.

## Results

Fourteen teachers were enrolled in the study (Table 1). All the participants were female with a median age of 33 with a range of 19 to 55. Most teachers (78.6%) were married, were educated at the graduate level (71.4%) and had more than 36 months of teaching experience (57.1%). The primary languages spoken amongst the participants were Sindhi (28.6%) and Balochi (71.4%). This was representative of the local community.

**Table 1:** Demographic information of the study participants

Variables	Frequency(*n* = 14)	Percentage (%)
**Marital Status**		
Married	11	78.6
Unmarried	3	21.4
**Language/ Ethnicity**		
Sindhi	4	28.6
Balochi	10	71.4
**Education Level**		
Intermediate	1	7.1
Graduate	10	71.4
Postgraduate	3	21.4
**Teaching experience in Deh Chohar School**		
Less than & equal to 6 months	2	14.3
7–12 months	1	7.1
13–24 months	1	7.1
25–36 months	2	14.3
More than 36 months	8	57.1

### Effect of training on the knowledge of teachers

To assess the effectiveness of each of the four modules on participants’ knowledge, a short assessment was conducted before and soon after each of the four modules training (Table 2). The only module that showed a significant difference (p value = 0.005) in knowledge was module 2 with a pre/post scores median (IQR) of 3(1) and 4(0.5). The rest of the modules (modules 1,3 and 4) showed no significant difference in knowledge (p value > 0.05).

**Table 2:** Pre and post scores of knowledge assessment

Assessments	Module 1		Module 2		Module 3		Module 4	
	Pre	Post	Pre	Post	Pre	Post	Pre	Post
	Median (IQR)	Median (IQR)	Median (IQR)	Median (IQR)	Median (IQR)	Median (IQR)	Median (IQR)	Median (IQR)
**Knowledge Scores**	3 (2)	4 (1)	3(1)	4(0.5)	3(1.25)	3(1.25)	4(3)	4 (2.5)
**p value**	0.071		0.005*		0.914		0.518	

*Wilcoxon Signed Rank Test significance p-value

### Effect of training on the resilience and self-efficacy of teachers

The dependent variables in this study were the General Self-Efficacy, and Resilience Scores. The Wilcoxon Signed Rank Test determined that significant difference from the pre to post module scores of GSE (p value = 0.016) and resilience (p value = 0.006). The pre median scores with IQR of the Self-Efficacy were 28(10), and Resilience scores 76(12) respectively; and post-score median score with (IQR) for the Self-Efficacy and Resilience scores increased to 35 (5.5) and 88 (14) respectively. This indicated a significant improvement in general self-efficacy and resilience skills after four weeks of training (Table 3).

**Table 3:** Pre and post scores of general self-efficacy and resilience

	Pre-Score (Median -IQR)	Post Score(Median -IQR)	p value
General Self Efficacy	28(10)	35 (5.5)	0.016*
Resilience	76 (12)	88 (14)	0.006*

*Wilcoxon Signed Rank Test significance p-value

### Qualitative feedback

In the qualitative feedback, most of the participants shared that sessions were very informative and useful. They gained new knowledge especially on effective communication, problem solving skills, psychological struggles of different age groups of children, behavior management within the classroom, and methods to build their own and student’s resilience. Participants stated that these sessions were so interesting that they changed their thoughts about life and the information gained was not only useful at a school level, but also at a personal level. Few participants suggested that they needed more refresher courses in the future with extended timelines.

## Discussion

These modules were initially planned to be implemented online. After discussion with the teachers, who felt that internet services were patchy and were more comfortable in person discussions, the workshops were converted to face-to-face sessions. Though it will be easy to do these sessions online, the efficacy might change. Additionally, these sessions had to be done during school time, as teachers complained of added stress if they had to take out additional time. This is something training teams need to be cognizant of, while planning workshops. The workshop itself should not become an extra burden.

The importance of a pre-intervention meeting was extremely important. It helped the team understand the struggles of the community that this school was a part of. This led to modification in some of the curriculum, bringing in relevant cases and interventions. Poverty, drug addiction, parental abandonment and violence were common struggles. Therefore, it is important that whenever such training is implemented, the social environment must be well understood.

The training team had made different case scenarios for each session, but teachers were hesitant to participate in role plays or discussions. Ultimately, the teachers were asked to share their own cases on that topic. This encouraged improved participation from the teachers. One teacher discussed the case of a child, who suddenly had academic decline. On further investigation, the teacher realized that the child’s father had been incarcerated and mother had abandoned the children. The teacher made efforts to contact the mother and was able to convince her to return. The teacher then proudly shared that this had resulted in the child improving. The other teachers became very involved in this discussion and gave more supportive suggestions, talked about their frustrations when dealing with such social difficulties and encouraged each other. Therefore, it is important that teachers be equal partners in these modules. Relevance helped improve participation. The team’s role was to help them discuss their cases, for all to learn from each other’s experiences, without fear, shame, or reprimand. It also helped build a supportive peer community, which could continue even after these sessions ended. Teachers felt more confident when they realized that their efforts were appreciated, and failures were accepted. Belonging to a supportive community and leaning on each other appeared to help teachers in their feeling of resilience and self-efficacy.

As teachers were taught about psychiatric disorders which led to learning difficulties, they were able to identify pathology in some of their students, who they had been struggling with. It was important to provide practical and relevant guidelines on how to help these children. In discussing a child with mathematical difficulties, a teacher was able to understand the child’s struggle rather than blame it on bad behavior or lack of effort. She was then able to go back to her class and worked on being more encouraging towards this child, complimenting his efforts, giving him small responsibilities, and providing additional help. Next session, this teacher proudly discussed how the child had improved in a quiz, and realized that despite his difficulties, a more understanding environment helped. This was an example of improving self-efficacy leading to creative solutions and success. These workshops, by providing a safe and supportive platform, also created an example for what needs to happen in classrooms.

Ultimately this was a learning experience all around. There were clear objectives but then there were hidden objectives, that came through the learning environment in these workshops. Basic elements of respect, equality, relevance was practiced and replicated. This level of interaction also helped build pride in the teachers at their own mastery of their skills, in very difficult situations, and encouraged them to learn further from each other. This might have been the biggest element that resulted in the significant improvement in resilience and self-efficacy scales in these teachers. This is an important element to remember for all training teams, who work in different scenarios from their own. Teaching in a hierarchical fashion alienates the subjects and decreases the efficacy of interventions. This is also a sustainable solution as builds their ability to work through problems that they face through their own skills and peer support.

The training team also learnt from the empathy of these teachers. A scenario was discussed, where a student stopped coming to school. On inquiry, they found out that she had no money for books. The teachers collected money and bought books for this child. A teacher also discussed a child with massive aggression, which despite different efforts, did not improve resulting in the child being asked to leave. The regret and guilt of failure was apparent in this teacher. It was heartening to see how all her peers came together to support and discuss limitations and failure despite effort: another lesson that can be expanded to students.

Modules on emotional awareness allowed teachers to discuss their home struggles. They shared different relaxation techniques, like praying, sitting outside, or talking to a family member. These were all very culturally relevant supportive techniques and the team realized that it was more effective to help teachers in finding time to practice these, rather than teaching breathing exercises or other commonly taught mindful exercises. Resilience improved by increasing emotional awareness and scheduling relevant relaxation techniques.

In summary, supportive school administration, scheduled peer support sessions, emotional awareness and support are helpful interventions to improve resilience in teachers. Recognition of effort and appreciation are helpful in improving motivation. Knowledge on relevant childhood disorders also helps improve self-efficacy and creativity in teachers.

### Limitations

One limitation of this study was that it was conducted in an NGO adopted school which might have helped encourage better participation by all teachers. Additionally, the teachers felt very supported by the NGO representative for this school. The representative was very well informed of all the difficulties that teachers faced. This was evident in the workshops as whenever a case scenario was discussed; the representative was already aware of those cases and had provided help to the teachers to find solutions. Therefore, the efficacy of these workshops might vary without the involvement of a supportive administrator from the school.

Additionally, there was a high percentage of higher trained teachers in this school, when compared to other government schools. Lastly this was a pilot study done on only 14 teachers, in one school and thus is not generalizable.

## Conclusion

In conclusion, the study revealed that building knowledge regarding mental health struggles in students and oneself, learning ways to cope with stress and manage student behavior and forming a peer support system are crucial in building self-efficacy and resilience in teachers. These are easy interventions to replicate for any country with a school system. They provide a structure and general themes of what should be covered but allow flexibility to fit the needs of different areas and cultures. This study needs to be conducted at a larger scale in multiple schools, to allow generalizability of findings. These results need to be followed longitudinally to look at long term impact. This study needs to be followed up with investigating student parameters and if they change once teachers’ resilience and self-efficacy improves.

## Data Availability

The dataset associated with this study can be made available on request from the corresponding author.
